# Concentrated Growth Factors (CGF) Combined with Melatonin in Guided Bone Regeneration (GBR): A Case Report

**DOI:** 10.3390/diagnostics12051257

**Published:** 2022-05-18

**Authors:** Alessandro Leonida, Gaia Favero, Paolo Caccianiga, Saverio Ceraulo, Luigi Fabrizio Rodella, Rita Rezzani, Gianluigi Caccianiga

**Affiliations:** 1 School of Medicine and Surgery, University of Milano-Bicocca, 20900 Monza, Italy; alessandro.leonida@unimib.it (A.L.); p.caccianiga@campus.unimib.it (P.C.); saverio.ceraulo@unimib.it (S.C.); gianluigi.caccianiga@unimib.it (G.C.); 2 Anatomy and Physiopathology Division, Department of Clinical and Experimental Sciences, University of Brescia, 25123 Brescia, Italy; luigi.rodella@unibs.it (L.F.R.); rita.rezzani@unibs.it (R.R.); 3 Interdipartimental University Center of Research “Adaption and Regeneration of Tissues and Organs—(ARTO)”, University of Brescia, 25123 Brescia, Italy

**Keywords:** concentrated growth factors, guided bone regeneration, melatonin, postoperative swelling

## Abstract

During implant restorative dentistry, common and crippling postoperative complications are pain and swelling of perioral soft tissues which engraving on patient quality of life. Concentrated growth factors (CGF), a novel generation of autologous platelet concentrate, and melatonin, endogenous indoleamine with also bone regenerative properties, may be useful for reconstruction of bony defects as well as in prosthetic and esthetic rehabilitation. We report a clinical case in which guided bone regeneration was performed combining CGF, melatonin and heterologous biomaterial. Great postoperative recovery without any complications was reported. In conclusion, in restorative dentistry the combined use of CGF and melatonin may have important roles in restoring bone defect, in improving implant osteointegration and, not less important, in preventing postoperative complications.

## 1. Introduction

The restoration of partially or total edentulous patients through guided bone regeneration (GBR) surgery combined with endosseous implants is actually the most diffuse dental treatment choice due to its predicable and solid outcomes [1,2,3,4]. However, the postoperative swelling of perioral soft tissues and the growth of ecchymosis or maxillary cyst still are common and debilitating complications. These postoperative inconveniences lead to difficulty in eating and sleeping [5,6,7], and so compromise the quality of life of patients.

From an oral health perspective, the autologous platelets concentrates appear to be very promising to fasten postoperative wound healing as well as to reduce the burden of postoperative complications [8,9,10]. Platelet-rich plasma was the first generation of platelet concentrates used in periodontal regeneration therapy and, to date, it is greatly used in oral and maxillofacial surgery [11,12,13,14]. Among the novel generation of platelets concentrates, Concentrated Growth Factor (CGF) obtained great attention for the application as a biocompatible regenerative material [15,16,17,18]. CGF is produced by centrifuging blood samples with a specific device that permits the isolation of a large and dense fibrin matrix rich in growth factors [19,20,21].

Melatonin, endogenously produced indoleamine, may target the overall bone remodeling process and promotes osteointegration [22,23,24]. Melatonin also has important effects against inflammation and oxidative stress processes [25,26,27], improving overall periodontal health.

The present case report assessed the clinical and radiological outcomes of combined use of CGF and melatonin in GBR surgery at the aim to restore bone deficiency and, not less important, to avoid postoperative common complications.

## 2. Case Report

**Figure 1:**
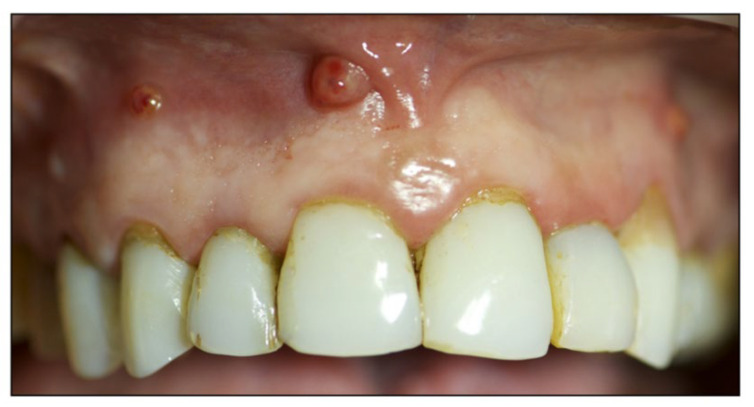
Intraoral preoperative examination.

We report a case of a 52-year-old healthy female patient presented recurring inflammatory process and multiple abscesses at the upper and lower jaw (Figure 1).

**Figure 2:**
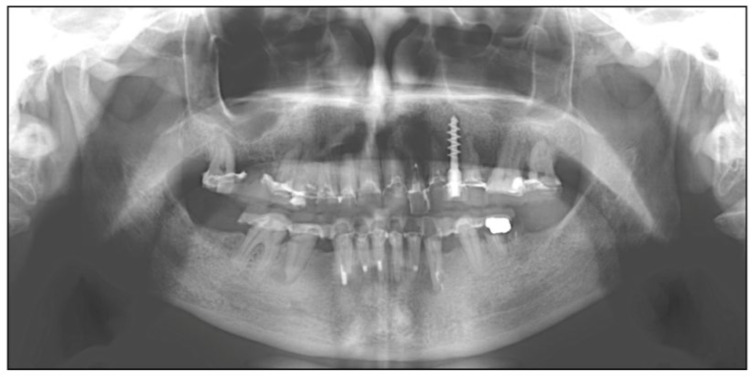
Preoperatory panoramic radiograph.

The patient showed mobility of the endosseous prosthetic devices at both dental arches due to substantial bone defect. The patient was a nonsmoker and had no systemic diseases that may prevent in proceeding with implant management. The panoramic preoperative radiograph showed various radiotransparent elements around almost all teeth of both upper and lower jaw (Figure 2).

**Figure 3:**
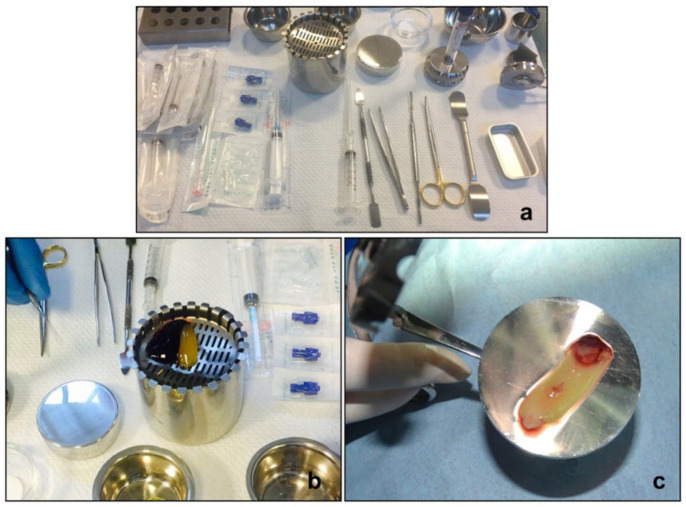
Concentrated growth factor (CGF) preparation. (**a**) Surgical instruments for CGF preparation; (**b**) CGF plus melatonin and (**c**) fibrin buffy coat pressed in a membrane.

Based on patient clinical and radiological observations, GBR surgery was planned to restore bone deficiency. Under local anesthesia, a mucoperiosteal flap was effectuated, then the teeth were extracted and a surgery debridement of both upper and lower jaw was performed. A significant bone lack due to a wide variety of infections was confirmed. At the time of surgery, a blood draw of the patient was performed (8 tubes–9 mL/tube). The CGF was isolated from the autologous blood samples, following standardized protocol. As we previously reported [21,28,29], the venous blood tubes were immediately centrifuged in Medifuge (Medifuge MF200—Silfradent Srl, Forlì, Italy) using the programme with the following characteristics: 30 s acceleration, 2 min at 2700 rpm, 4 min at 2400 rpm, 4 min at 2700 rpm, 3 min at 3000 rpm, and 36 s deceleration and stopped. At the end of the centrifugation process, the CGF was obtained (Figure 3).

With respect to the standardized protocol reported, each CGF tube also contained 0.0232 mg of powered pure synthetic melatonin.

**Figure 4:**
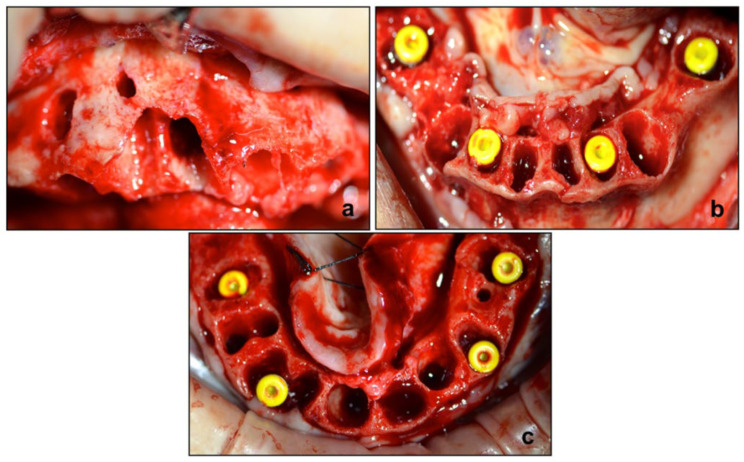
Intraoperative procedure. (**a**) Site preparation for Guided Bone Regeneration procedure and Max Stability implants positioned in the upper (**b**) and lower (**c**) jaw.

After regularizing the alveolar ridge, four Max Stability implants (Leone, Florence, Italy) were inserted contextually in the upper and lower jaw (Figure 4) for Toronto-bridge prosthesis. The CGF red component was separated by fibrin buffy coat and blended with OX granular biomaterial (Bioteck S.p.A., Vicenza, Italy).

**Figure 5:**
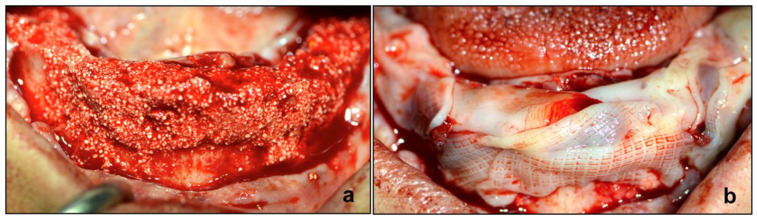
Bone graft and concentrated growth factor (CGF) combined with melatonin. (**a**) Biomaterial blended with CGF red component in situ and (**b**) CGF membrane plus melatonin in situ.

The autologous fibrin buffy coat was pressed and transformed into a membrane located in both dental arches (Figure 5). The surgical flap was sutured with 4.0 silk suture and, finally, to protect the surgical-treated zone, the autologous serum was used.

Postoperative care included antibiotic (amoxicillin 1 g/die) for 6 days and nonsteroideal analgesics for 7 days. The patient has followed a soft-food diet for about one month.

**Figure 6:**
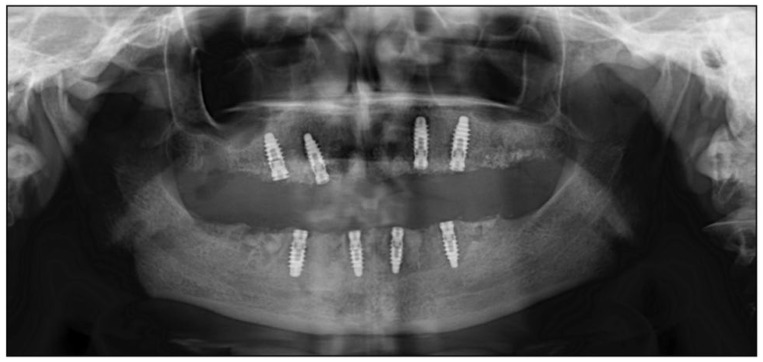
Postoperative (5 days after surgery) panoramic radiographs.

The clinical and radiographic evaluations 5 days after surgery revealed significant bone regeneration. In detail, the surgical-treated area showed appropriate bone density and volume that permitted great implants stability (Figure 6).

Interestingly, there were no infectious episodes and no other adverse complications during the monitoring postoperative period. The postoperative healing response at the surgical-treated sites was excellent, and the patient reported a good recovery without discomfort or inconvenience. Obviously, the absence of common surgery complications coincided with no difficulty in postoperative course without encroaching on patient quality of life.

## 3. Discussion

This case report presents a clinical case in which tissue engineering technique succeeded in treating osseous defects; in fact, melatonin in combination with autologous CGF were successfully used in the GBR treatment of bony defect to achieve favorable clinical results. We demonstrated that this novel regenerating approach might have great potential to improve clinical and radiographic parameters and in preserving the patient quality of life. Improve surgery techniques and patient postoperative outcomes are essential to prevent altered bone regeneration and implant loss.

PRP was found to be successful in achieving bone regeneration, but due to its limited properties (like poor handling characteristics or immunogenicity [30]), CGF and second generation of platelet concentrates are founding wide application in the regenerative therapy. Unlike PRP, CGF does not dissolve rapidly and, due to the presence of a large number of platelets in the fibrin network, the release of growth factors is slowly and cover a 14-day period [31]. Thus, CGF could be considered a powerful bio-scaffold with a great reservoir of growth factors and with a poor incidence of surgical complications and morbidity. Furthermore, the absence of additive molecules during its preparation is another fundamental benefit for the use of CGF [21,32,33,34].

The present clinical study showed that the use of CGF combined with melatonin has an important role in restoring stable bone volume around dental implants and, not to be underestimated, prevented swelling and pain leading to a comfortable postoperative course.

In agreement with our observations, Mirković et al. [35] reported that CGF, alone or mixed with bone graft, can be useful for reconstruction/regeneration of bone defects. It is important also to emphasized that CGF may contribute to reduce postoperative edema and implant relapse, without presenting risk of transmissible and allergic diseases and it is also cost-effective [35,36]. Our findings are in accordance with Dai et al. [32] retrospective study, in which GBR was applied with the combined use of mineralized collagen showing gratifying effects not only in shorten the soft tissue healing time, but also in alleviate the postoperative discomforts. However, the Authors not evaluated the CGF effect on bone regeneration. In our clinical case the patient reported irrelevant postoperative pain and swelling; whereas, Dai et al. [32] described that patients suffered of swelling, pain, chewing impairment and nausea on the first day after surgery. Yu et al. [37] observed in patients with single maxillary anterior teeth loss and labial orbital bone defect that CGF, respect collagen membrane, significantly reduced postoperative swelling, but was not significantly effective against pain. Notably, the clinical results of the present case report were even more favorable than clinical outcomes reported by Yu et al. [37] and Dai et al. [32]. Recently, Taschieri et al. [38] performed a prospective comparative study comparing standard implant treatment to the combined application of implants and CGF at the aim to determine the effect of CGF on quality of life. In agreement with our observation, the Authors reported that CGF positively influenced patient quality of life when associated with implant rehabilitation of mandibular molars, minimizing post-operative discomfort. A fundamental factor that should be considered in interpreting our satisfactory clinical outcomes is the combination of CGF with melatonin, underlining their synergic effects. It has been demonstrated that melatonin has an anti-edema action [39,40] and also analgesic effect [41]. Remarkably, to date no study has reported serious adverse effects correlated to exogenous melatonin applications [42].

## 4. Conclusions

To our knowledge, this is the first clinical report that used CGF plus melatonin-based scaffold in GBR surgery holding a promising outcome in tissue regeneration applications and restorative dentistry. In the future, randomized controlled studies involving greater number of patients and monitoring them for a long postoperative period are necessary to definitely demonstrate the successfully result(s) that we reported in the present case report. Furthermore, it will also be important to investigate the interindividual predisposition factors involved in the process of prosthetic and esthetic rehabilitation in depth.
